# Cognitive adaptation in asexual and sexual wasps living in contrasted environments

**DOI:** 10.1371/journal.pone.0177581

**Published:** 2017-05-12

**Authors:** Lucie Froissart, Martin Giurfa, Sandrine Sauzet, Emmanuel Desouhant

**Affiliations:** 1 Laboratoire de Biométrie et Biologie Évolutive UMR 5558, Université Claude Bernard, Université de Lyon, CNRS, Villeurbanne, France; 2 Institut Universitaire de Technologie Lyon 2, Université Lumière Lyon 2, Université de Lyon, Bron, France; 3 Research Center on Animal Cognition, Université de Toulouse, CNRS, Toulouse, France; Biomedical Sciences Research Center Alexander Fleming, GREECE

## Abstract

Differences in learning and memory dynamics between populations are suspected to result from differences in ecological constraints such as resource distribution. The two reproductive modes (strains) of the parasitoid wasp *Venturia canescens* share the same geographical areas but live in contrasting habitats: arrhenotokous wasps live in the wild (generally orchards), whereas thelytokous ones live mostly in stored-products buildings (*e*.*g*. granaries). This species thus represents a relevant biological model for understanding the relationship between the ecological constraints faced by a species and its memory and learning ability. We showed that after having laid eggs in presence of both a synthetic odour and natural olfactory cues of their host, arrhenotokous wasps exhibited a change in their behavioural response towards the synthetic odour that was at least as pronounced as in thelytokous ones even though they were faster in their decision-making process. This is consistent with better learning skills in arrhenotokous wasps. The corresponding memory trace persisted in both strains for at least 51 h. We compare and discuss the learning and memory ablities of both strains as a function of their costs and benefits in their preferential habitats.

## Introduction

Most animals forage for patchily distributed resources in fluctuating environments. Learning is a process allowing to acquire information on resource availability and distribution and which thus reduces the uncertainty of the environment [1]. Similarities in the basic traits that define learning and memory are shared between phylogenetically distant animals, from the existence of similar associative processes and memory phases [2], to commonalities in the neural and genetic pathways underlying these processes [3–5]. However, differences in specific learning and memory features exist between closely related taxa, such as in the case of memory duration [6–9]. Such differences may reflect ecological and evolutionary differences between related taxa and may constitute, therefore, species-specific adaptations to different lifestyles [10].

The idea that ecological constraints shape memory and learning features is supported by different approaches. First, in the honeybee *Apis mellifera* the dynamics of memory phases is correlated with the dynamics of foraging cycles, with short travels between flowers inside a patch in the range of seconds corresponding to short-term memories and foraging bouts occurring after days, weeks or overwintering months, to stabilized long-term memories [10]. Second, theoretical works also predict that the speed of changes or the spatial distribution of resources should influence the duration of information retention in memory (*e*.*g*. [11–13]): fast changes favor short lasting memories, spatial heterogeneity promotes longer memory traces. At last, comparative studies between populations or closely related species that do not share the same environment allow testing whether environmental constraints on foraging can be correlated with learning and memory differences. For example, food-caching birds facing limited and unpredictable food supply and whose survival hangs on accurate food-cache recovery display better spatial memory than birds of the same species living in milder environments [14].

Parasitoid insects, whose larvae develop at a host’s expense, generally another arthropod, are relevant for these comparative studies [4]. These insects represent a highly ecologically diverse group [15] and show remarkable learning abilities (*e*.*g*. [16–18]). Moreover, in the context of associative learning, natural variations in learning and memory traits were highlighted between species, as in *Nasonia* spp. (*e*.*g*. *N*. *vitripennis*, *N*. *giraulti*: [19]). Another series of studies examined the associative learning ability and memory dynamics of two closely related species of the genus *Cotesia* and suggested that adaptation to different host distributions led to different memory dynamics. While *C*. *glomerata*, which parasites aggregated hosts in homogeneous environments, is able to form a memory lasting at least 5 days after one single host-plant association encounter, *C*. *rubecula*, which meets scattered hosts in heterogeneous environments, needs several host-plant association encounters to build a long term memory ([8, 20]). Although these studies focused on the interspecific level, one could expect variations in learning ability and memory dynamics at the intraspecific level if populations live under different environmental conditions and thus are submitted to different selective pressures.

*V*. *canescens* is of particular interest because it offers the opportunity to compare learning in two strains that live in contrasting environments. In this species, two reproductive modes are found: arrhenotoky, where males arise from unfertilised eggs and females from fertilised eggs, and thelytoky, that corresponds to obligate parthenogenesis that produces only females. Individuals of both reproductive modes share the same geographical areas [21, 22], but live in distinct preferential habitats. Arrhenotokous wasps live exclusively outdoors, in orchards [22, 23] whereas thelytokous wasps are mostly found in stored-product buildings, such as mills or granaries [22, 23]. Although females of each reproductive mode are indistinguishable to human eyes, they differ in a number of phenotypic traits, correlating with the divergent selective pressures they experience (*e*.*g*. egg production and energy allocation: [24]; information use and host patch exploitation: [25–27], genetic variability: [28], phenotypic plasticity: [29]).

In outdoor conditions, arrhenotokous wasps face a low-density host distribution: host larvae are concealed in fruits, with each infested fruit holding only one or two larvae [30]. In thelytokous wasps’ indoor environments, hosts can reach much higher densities than in the wild and tend to aggregate in large patches [31]. Two main differences in host-seeking resulting from these different distributions can be outlined. First, in the wild, wasps have to fly from host to host because they are widely scattered in the environment, whereas indoors, a wasp walking inside a patch can find numerous hosts in close vicinity. Indoor flights are limited to travels between large patches. Host-seeking in the wild therefore implies a higher investment in flight, an energetically costly behaviour [32]. Second, when searching for hosts while flying, both wasp strains can rely on a volatile secretion produced by host larvae (a kairomone) which innately attracts these parasitoids [33]. Although this cue of host presence should be of comparable reliability in both habitats, the detectability of outdoor solitary larvae based on the presence of this cue should be lower compared to indoor host patches, due to the comparatively higher amount of kairomone produced by aggregated larvae in the latter situation [34]. Environmental cues associated with host presence and easily perceptible at a distance, like olfactory cues related to the host feeding substrate, should enhance the detectability of larvae in outdoor habitat [35], but would only be redundant with host kairomone in indoor habitat. Learning such environmental cues should then decrease flight time in larger proportions in arrhenotokous wasps. Since there is also a longer flight time to save in those wasps, learning environmental cues associated with host presence should result in a higher fitness gain in arrhenotokous wasps due to a greater time-, energy- and risk-saving [36], and should be selected for in that strain.

According to the host distributions, arrhenotokous wasps should then have evolved better skills at learning new indirect cues of host presence than thelytokous ones. This should translate into a greater behavioural plasticity, that is a more pronounced change in behaviour, in arrhenotokous wasps, allowing them to come back more easily to cues encountered in association with hosts. From an evolutionary perspective, the duration of memory resulting from that learning process should depend on the balance between the value of information—that is, the benefit of learning [37]—and the cost of storing information, which can be physiological [38] or ecological (the cost of using wrong or out-of-date information; [39]). Assessing this balance is not straightforward and prevents predictions of which strain should retain information for a longer time.

Here we evaluated whether different learning abilities and memory durations evolved in the two strains of *V*. *canescens* by testing wasps after several generations of rearing under the same lab conditions. The wasps were trained to associate an artificial odour and host presence, a procedure classically used in the study of parasitoid learning (*e*.*g*. [4]).

## Materials and methods

### Biological material

*Venturia canescens* is an endoparasitoid whose females parasitize lepidopteran larvae, mainly Pyralidae [40, 41]. *V*. *canescens* females search for hosts that are concealed in the substrate, such as stored products or fruits, by probing the host-exploited substrate with their ovipositor. The females respond innately to a mandibular gland secretion that the host deposits in its food substrate [42]. These chemical acts as a pheromone and mediates host population regulation. But they also act as a kairomone that confers a benefit only for the receivers (*i*.*e*. the parasitoids). In addition to guiding parasitoids to hosts at a distance, this volatile substance elicits oviposition behaviours (drumming the host-exploited substrate with antennae and probing into it with the ovipositor). If the host-exploited substrate and then the kairomone are removed, a wasp does not try to oviposit even in the presence of host larvae (L.F. personal observation). Thus, the kairomone triggers a series of innate behaviours implied in host detection and oviposition. Oviposition is recognized based on a characteristic movement of the abdomen called ‘cocking’, which follows egg-laying [43, 44]. Previous studies showed that *V*. *canescens* learns olfactory and visual cues in the contexts of host- and food-searching, respectively (*e*.*g*. [45, 46]).

In our experiment, we used two strains of wasps (one of each reproductive mode) originated from individuals captured in two sites 12 km far from each other near Valence, France: an organic orchard (cherries and apples) in Gotheron (GPS coordinates: N44 58.344 E4 55.659, Institut National de la Recherche Agronomique (INRA)), with the permission of Vincent Mercier from INRA and hedges near a grain silo in Montélier (N44 56.300 E5 00.115). No permission was required in the latter case, since sampling occurred in a public area. *Venturia canescens* is moreover not an endangered or protected species. This large field sampling was conducted in summer 2008. The reproductive mode of each sampled individual was assessed in the lab based on the sex-ratio of their progeny. In each strain, the initial laboratory population was set from 70 individuals. The wasps were reared on *Ephestia kuehniella* Zeller (Lepidoptera: Pyralidae) larvae, a pest of stored products. Host larvae were obtained from a mass-rearing facility (Biotop, Antibes, France) and grown on wheat semolina. *Venturia canescens* females parasitise *E*. *kuehniella* larvae in the second to the fifth instar [40]. The parasitoids and hosts were kept in a controlled environment (rearing conditions: 25±1°C, 75±5% relative humidity and 12:12 h light:dark).

The experiment was conducted in July 2010 after 30 generations of rearing both strains under the same lab conditions. This excludes the possibility to highlight differences due to individual experience in a given environment, or due to maternal effects. For the experiments, the wasps were isolated immediately after their adult emergence in a plastic tube (30 mm diameter, 70 mm height). They were provided with water and fed with 50% water-diluted honey 24 h after emergence. When not manipulated, all wasps were kept under the rearing conditions in a room without any host odour, or chemicals used during the experiment (see below). The reproductive mode of each individual tested was checked a posteriori through the sex-ratio of its progeny.

The experiment performed complies with French law about animal care and experimentation. Insects were killed by freezing at the end of the experiment.

### Learning ability and memory duration

#### Overview

**Fig 1:**
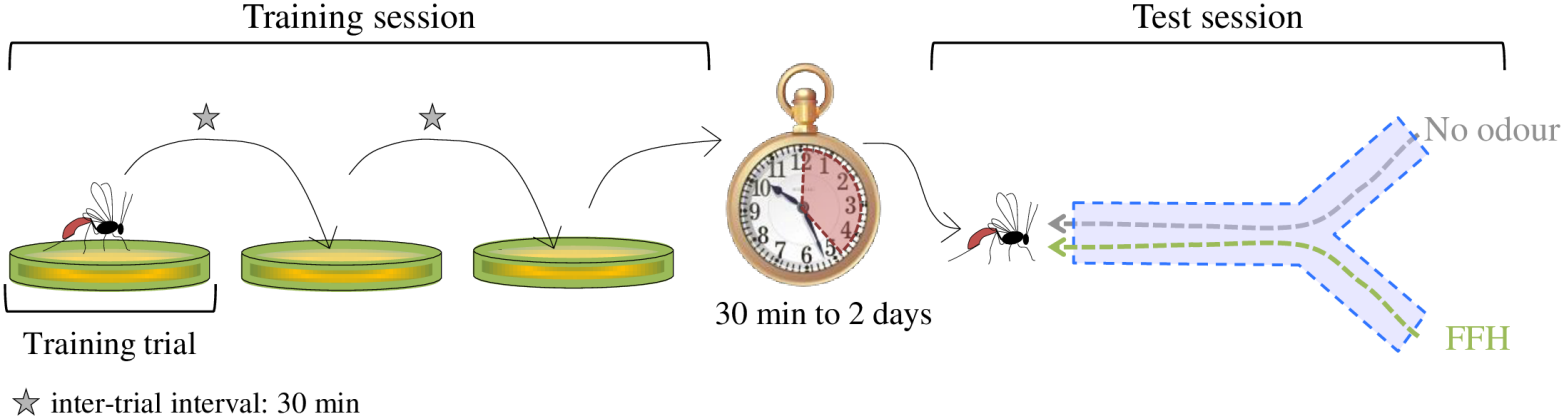
Wasp’s training and testing: Overview. A training trial consisted in allowing a wasp to oviposit once in the presence of host kairomone and the training odour.

We compared the memory retention established in the two strains after a training session (Fig 1, see section “Training session” for the details). Each trained wasp was individually exposed to a synthetic odour (hereafter called the training odour) simultaneously with host kairomone and allowed to oviposit. It was afterwards subjected to a test session (Fig 1, see section “Test session” for the details) in which we recorded its choice of the training odour *vs*. no odour, in the absence of host kairomone. To investigate if the training session resulted in a change in the choice behaviour, the response of trained individuals was compared to that of naïve wasps that had neither encountered the training odour, nor host kairomone. This comparison was conducted at different time intervals after training (from 30 min to 2 days, Fig 1), each individual being tested alone and at only one time interval.

#### Training session

The day after emergence, females of each strain were randomly assigned to 2 groups: “naïve” (control) and “trained”. The trained wasps were submitted to a “training session”.

The training session consisted of three “training trials” spaced 30 min apart. Such spaced training is known to favour long-term memory formation in Hymenoptera [8, 47]. During a training trial, a single wasp was deposited on a “training patch”, where it was allowed to oviposit once in the presence of both host kairomone and the training odour. A solution of furfuryl heptanoate (FFH, Sigma-Aldrich, Saint-Quentin-Fallavier, France; purity 98%) diluted at the rate of 1% V/V in ethanol (purity 96%) was chosen as the training odour. This non-natural and synthetic odourant molecule has been previously used successfully in classical conditioning experiments on the parasitoid *Lariophagus distinguendus* [48].

A training patch was created by laying 100 fourth-instar host larvae (without food medium and hence without host kairomone) on a plastic Petri dish (5 mm deep, 55 mm diameter) that was covered by a thin mesh to prevent the larvae from escaping. 5 μL of FFH solution (*i*.*e*. the training odour) and 200 μL of a host kairomone solution were deposited on two other meshes, tightened on the first one. The host kairomone solution was obtained by washing host-exploited semolina with a 50% acetone and 50% ethanol solution (adapted from [42]). The solution, that is attractive and triggers oviposition attempts in *V*. *canescens* females [33], as do natural host patches and pure kairomone [42], was deposited the day before the experiment and left to dry for approximately 24 h. A new training patch was used every 30 min (a given wasp never oviposited twice in the same patch, but 12 to 15 individual trainings happened successively on the same patch).

The trained wasp left its emergence tube on its own being attracted by kairomone. Once on the training patch, in most of the cases, it immediately drummed its surface with its antennae and started probing the layers with its ovipositor. Because of the high number of host larvae in the training patch, oviposition usually occurred within a few seconds after the beginning of probing (approximately 10 sec). Any wasp that did not cock within 3 min was discarded. Given that arrhenotokous females lay eggs at a lower rate than thelytokous ones [27], more trained arrhenotokous (37% out of 261) than trained thelytokous wasps (11% out of 270) were discarded. Each wasp was allowed to walk back to its emergence tube once the cocking movement happened, indicating oviposition. Four arrhenotokous and 11 thelytokous trained wasps died before the testing session start.

Arrhenotokous wasps spent in average (+SE) 51.13 s (+1.49 s, *N* = 133) on each training patch while thelytokous wasps spent 45.33 s (+1.09 s, *N* = 126). This small difference (Cohen’s *d* +95% confidence limit = 0.24+0.14) is not biologically relevant to account for behavioural differences in the test session.

The training trials were performed inside a plastic-net box (30x30x30 cm) under the same temperature and humidity conditions as rearing. The training trial of an arrhenotokous wasp was followed by one of a thelytokous wasp. Each day, the first training trial of the first trained wasp began between 10:00 AM and 10:30 AM and the third training trial of the last trained wasp ended between 11:30 and 12:00 AM.

#### Test session

During the test session, each wasp of the trained group had the choice between the training odour and no odour in an olfactometer (see below). The behaviour of each wasp was assessed once at one given time interval after their last training trial.

The olfactometer was a glass Y-shaped tube (foot length: 24 cm, arm length: 11 cm, angle between arms: 80°, inner diameter: 35 mm; see [49]). Each arm of the tube was connected to a “target chamber”, a 7-cm glass tube in which odour sources could be placed. A fan produced laminar airflow from the arms to the base at a speed of 20 cm.s^-1^. The air was filtered through activated charcoal. Lighting came from a daylight tube that was placed beyond and 90 cm above the target chambers. To ensure an environment devoid of visual cues that could attract the wasps, the olfactometer was placed in the center of a white-painted wooden box (200x140 cm and 70 cm high).

Ten pieces of 5-mm diameter filter paper fixed with a needle hanging on a plastic cork were placed in each target chamber. On one side of the olfactometer, each piece of filter paper was soaked with 5 μL of FFH solution (hereafter called “training odour”). On the other side, the pieces of filter paper were left without FFH or solvent (hereafter called “no odour”). The olfactometer was cleaned with water and detergent (Microson, Fisher Scientific) and the filter papers renewed at most every 1.5 h (at most each 15 tested wasps) to remove potential chemical marks left by females in the Y tube. This cleaning procedure and the random alternation of trained and naïve wasps in the olfactometer represents a conservative approach. Indeed an effect of a potential chemical mark should at worst buffer the difference due to treatment. The position of each target chamber was randomized each day to control for a potential side effect.

The tested wasp was individually introduced into the olfactometer through a hole near the base. A wasp was considered to have made a choice when it reached one target chamber. The choice of the wasp (training odour *vs*. no odour) as well as the time needed to choose were recorded. Wasps that did not move within 3 min or did not reach a target chamber within 5 min were discarded. All of the tests were conducted between 11:30 AM and 04:00 PM under the same temperature and humidity conditions as rearing. Preliminary experiments ensured that for both strains, the wasps’ choice was random in the olfactometer in the absence of odour.

**Fig 2:**
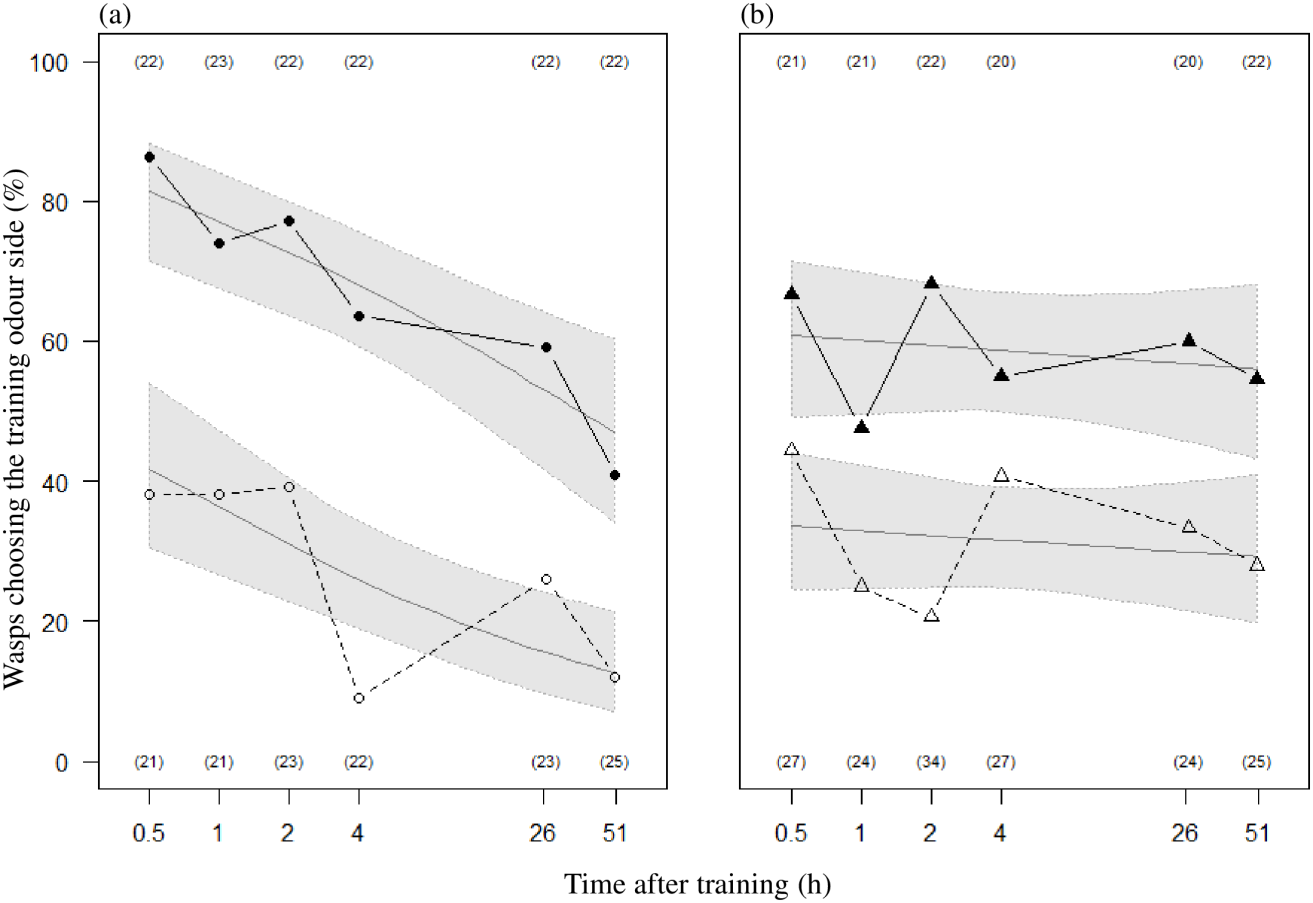
Proportion of females choosing the training odour at different times after a training session. Trained wasps were allowed to oviposit in the presence of FFH solution and host kairomones in three training trials spaced 30 min apart. Naïve wasps that did not experience such a treatment were tested at the same time and day. Each wasp was only tested once at a given time interval after its last training trial. (a) Arrhenotokous wasps. (b) Thelytokous wasps. Open symbols: naïve wasps. Full symbols: trained wasps. Sample sizes are indicated in brackets at the top (trained wasps) and bottom (naïve wasps) of each panel. The time after training is represented according to a logarithmic scale along the x axis. Since this variable is partly correlated with the time of the day and age (see Material and methods), its effect in trained individuals can only be evaluated through the evolution of the difference between trained and naïve individuals. The grey curves represent the predictions of the statistical model in each treatment. The grey shadows represent the corresponding 95% confidence interval of the predictions.

Wasps were tested for their response to the training odour 30 min, 1 h, 2 h, 4 h (± 2 min), 26 h or 51 h (± 20 min) after the last training trial (Figs 1 and 2). Each wasp was tested only once in the olfactometer. Because the last training trial happened always between 11:00 and 12:00 retention tests in the Y tube at a given time interval were always performed in the same time window (*e*.*g*. 1-h tested wasps were tested between 12:00 AM and 01:00 PM, and 4-h tested wasps were tested between 15:00 and 16:00 according to the exact timing of their last training trial). Therefore, time of the day and age of the trained wasps were positively correlated with the time interval after training, and may constitute confounding factors for this variable. As a consequence, the effect of the time after training can properly be evaluated only through the comparison of naïve and trained wasps (see “Data analysis” section). Training sessions were planed on series of 5 or 6 successive days, two consecutive series being separated by a break of one or two days, so that the response of the wasps of both strains from all time intervals after training could be assessed each day.

#### Control: Naïve wasps

As a control, the response of individuals that had neither encountered the training odour, nor host kairomone were tested in the olfactometer. Each of these naïve wasps was tested immediately before or after (±10 min) a same-aged trained wasp of the same strain. This allows to determine (1) whether the wasps are innately attracted, indifferent or repulsed by the training odour and (2) whether that behaviour varies in the course of the time of the day (or with age). In other words, it allows to determine the baseline behaviour. Then, any behavioural plasticity due to training can be detected by comparing the response towards the training odour of the trained with that of the naïve individuals, whatever the innate response (positive, neutral or negative) or the variation in the course of the day (or with age).

In total, 775 wasps were tested: 161 naïve and 161 trained arrhenotokous, and 225 naïve and 228 trained thelytokous wasps. Among these wasps, for each time interval after training, 21–25 naïve (that is 81–89% of the naïve arrhenotokous wasps tested) and 22–23 trained (73–88%) arrhenotokous females and 24–34 naïve (58–87%) naïve and 20–22 trained (50–66%) thelytokous females were considered to have made a choice.

### Data analysis

The response variable that we analysed as a proxy of memory retention following learning was the side chosen by the wasp in the olfactometer (*choice outcome*: training odour *vs*. no odour).

According to the predictions of a speed-accuracy trade-off [50], any difference in choice behaviour can be attributed to the speed at which it is made: slower individuals are expected to make the more accurate choices. Then, the time needed to reach one of the target chambers (*choice duration*) was also analysed to determine if differences in choice behaviour between the strains could be attributed to differences in the speed of the choice.

The *choice outcome* was analysed using generalised linear models (binomial distribution of error, logit link). The explanatory variables were the *strain*, the *training treatment* (naïve *vs*. trained), the *time after training* (0.5, 1, 2, 4, 26 or 51 h). Since data points were not evenly spaced according to *time after training*, and to avoid leverage by data points obtained at 26 and 51 h, this variable was included in the model after log-transformation. All the interactions between these variables up to the triple interaction were considered: (1) the analysis of the interaction between *time after training* and *training treatment* allowed us to seek whether the choice behaviour varies with *time after training* in a similar manner between naïve and trained wasps. It then allowed to control in trained wasps by comparison with naïve ones, the effect of age and time of the day (that correlates with *time after training*, see Material and method, Test session). (2) The triple interaction allowed to seek for a difference in memory duration between the two strains: it tests whether the choice behaviour difference between naïve and trained individuals changed similarly in the course of time in both strains.

To determine if the *choice outcome* differed from a random 50–50 choice, that is if the wasps were attracted, repulsed or indifferent to the training odour, we performed *χ*^2^ goodness-of-fit tests in each group of wasps, on the data pooled from all time points after training.

The *choice duration* was analysed by means of a generalised linear model with a Gamma distribution of error (inverse link) and explained by the *training treatment*, the *strain* and their interaction.

All statistical analyses and the figure were performed with the software R 3.0.3 ([51]; libraries: faraway, lattice and effects). All the variables and interactions were introduced sequentially. Non-significant effects were removed from the models through backward selection.

## Results

### Training modifies the wasps’ response towards the training odour

The response of the wasps towards the training odour was found to be plastic following the training, since *choice outcome* was influenced by *training treatment* (Table 1 and Fig 2): the percentage of individuals choosing the training odour was higher in trained than in naïve individuals.

**Table 1:** Analysis of choice outcome (training odour *vs*. no odour).

	*df*	Deviance	*P*>|*χ^2^*|
*strain*	1	0.53	0.46
*training treatment*	1	63.44	<0.0001
*time after training*	1	10.39	0.001
*strain x time after training*	1	5.96	0.015
*training treatment x time after training*	NS		
*strain x training treatment*	1	3.33	0.068
*strain x training treatment x time after training*	NS		

Generalised linear model (binomial distribution of error, logit link).

NS: non-significant, corresponding to factors excluded during the model selection procedure. We keep the interaction between *strain* and *training treatment* in the final model because it leads to the best model, that is the model with the lowest Akaike’s Information Criterion (AIC = 692.28). The second best model (ΔAIC = 1.33) includes the same explanatory variables as the best model but excludes the interaction between *strain* and *training treatment*.

Overall, naïve individuals from both strains were found to avoid the training odour (27% out of 135 arrhenotokous wasps chose the training odour, Chi-square goodness-of-fit test: $\chi_{1}^{2}=29.4$, *P* < 0.0001; 32% out of 161 thelytokous wasps chose the training odour, Chi-square goodness-of-fit test: $\chi_{1}^{2}=21.62$, *P* < 0.0001). Trained wasps prefered the training odour over no odour (67% out of 133 arrhenotokous wasps chose the training odour, Chi-square goodness-of-fit test: $\chi_{1}^{2}=15.22$, *P* < 0.0001; 59% out of 126 thelytokous wasps chose the training odour, Chi-square goodness-of-fit test: $\chi_{1}^{2}=21.62$, *P* = 0.05).

Trained wasps of both strains were slower to choose than naïve ones in the olfactometer (Table 2, effect of *training treatment*; naïve females: mean *choice duration* +SE = 105+4 s, *N* = 296; trained females: mean *choice duration* +SE = 139+4 s, *N* = 259).

**Table 2:** Analysis of choice duration.

	*df*	Deviance	*P*>|*χ^2^*|
*strain*	1	8.06	<0.0001
*training treatment*	1	11.83	<0.0001
*strain x training treatment*	NS		

Generalised linear model (Gamma distribution of error, inverse link).

NS: non-significant, corresponding to factors excluded during the model selection procedure.

### Arrhenotokous wasps choose faster, but the choice response is as plastic in both strains

Arrhenotokous females, both naïve and trained, chose faster in the olfactometer than thelytokous ones (Table 2, effect of *strain*; arrhenotokous females: mean *choice duration* +SE = 106+4 s, *N* = 268; thelytokous females: mean *choice duration* +SE = 135+4 s, *N* = 287). This result is consistent with the fact that more arrhenotokous than thelytokous wasps were considered to have made a choice after 5 min in the olfactometer.

The plasticity of the choice response is equivalent in both strains (Table 1, effect of *strain* x *training treatment*; Fig 2), even though naïve arrhenotokous wasps tend to choose the training odour less than naïve thelytokous wasps, and trained arrhenotokous wasps tend to choose the training odour more than trained thelytokous wasps.

### The effect of training persists up to 51 h in both strains

The difference between the percentage of naïve and trained individuals moving towards the training odour evolved in the same way in arrhenotokous and thelytokous wasps in the course of *time after training* (Table 1, *strain* x *training treatment* x *time after training*: NS). This difference between naïve and trained wasps persisted from 30 min to 51 h after training (Table 1, *training treatment* x *time after training*: NS). In other words, in both strains, the plasticity of the choice response following training did neither vanish nor was it weakened 51 h later.

### Arrhenotokous wasps decrease their choice of the training odour along the time of the day and/or with age

Considering the absolute response of the whole group of arrhenotokous wasps, and not the difference between the response of naïve and the one of trained arrhenotokous wasps (preceding paragraph), it appeared that the percentage of arrhenotokous wasps choosing the training odour globally decreased with the *time after training*; this was not the case in thelytokous wasps (Table 1, effect of *time after training*, *strain* x *time after training*; coefficient +95% confidence limit of (log-transformed) *time after training* in arrhenotokous wasps = -0.34+0.18, in thelytokous wasps = -0.04+0.22; Fig 2). But the *time after training* cannot explain that decrease in naïve individuals. However the time of the day at which the wasps were tested and their age, both correlated with *time after training* (see Material and methods, Test session) could account for this decrease. The percentage of trained arrhenotokous wasps choosing the training odour decreased at the same rate as that of naïve arrhenotokous wasps (Table 1, *strain* x *training treatment* x *time after training*: NS, *training treatment* x *time after training*: NS): thus, the decrease in training odour choice observed in trained arrhenotokous wasps could also be attributed to an effect of age and/or time of the day, and not to a decay of the effect of training.

## Discussion

We showed that the response towards an odour is modified after its association with a host kairomone in an oviposition context in both *Venturia canescens* strains: naïve females avoided the odour while trained females were attracted to it. This behavioural difference shows memory formation in trained wasps. It was as pronounced in both strains even if arrhenotokous wasps made their decision faster. These results suggest that arrhenotokous wasps learn more efficiently about odours than thelytokous ones in an oviposition context. Memory persisted in both strains up to at least 51 h.

Naïve individuals of both strains differed in their choice behaviour. While naïve thelytokous wasps choose the training odour at a stable rate, a decrease was found in naïve arrhenotokous wasps along the day (and/or with age). The same pattern was found in trained individuals (Fig 2). This difference was not an artefact linked to differences in the conditions under which the animals were tested as wasps of both strains were tested in parallel and in the same experimental conditions. Nevertheless, we cannot exclude an effect of an environmental variable varying with the time of the day (such as atmospheric pressure), to which only arrhenotokous wasps would be particularly sensitive. An effect of age is unlikely as all wasps tested were young (the 51 h-tested wasps were 3 days old) in regard of their life expectancy, which is about 1 month [49]. Thus they were not senescent. Further experiments are required to uncover to what extent the behavioural pattern observed in arrhenotokous wasps can be generalised and whether it depends on circadian factors [52].

Trained wasps of both strains preferred the training odour over no odour. On the contrary, naïve individuals of both strains, which never experienced host kairomone, oviposition or the training odour, avoided the training odour (Fig 2). This behavioural difference can be attributed to the retention of olfactory information following a learning process. Indeed, other processes that could explain such a preference shift [53, 54] can be excluded. For example, the training odour could impair the animal’s olfactory system, as some pesticides do (*e*.*g*. [55]). In such a case, the innate avoidance of the training odour should be reduced at best to a random choice, but this could not explain preference inversion. The behavioural plasticity could also be explained by a sensitizing effect of host kairomone and oviposition [56]. But individuals that were allowed to oviposit in the absence of the training odour (S1 Supporting information) chose the training odour at a lower rate than trained individuals. Thus, oviposition and/or the exposition to host kairomone alone cannot explain the behavioural plasticity evinced the trained wasps.

Arrhenotokous wasps made decisions of better quality, which suggests, in agreement with our predictions, better learning skills in this strain. Arrhenotokous wasps were faster to choose than thelytokous ones. This higher choice speed should have conspire against their choice accuracy [50]. But arrhenotokous wasps, both naïve and trained, were at least as accurate as thelytokous ones. Their higher speed then indicates that arrhenotokous wasps made decisions of better quality. This cannot be linked to a difference between the strains regarding the value of the reward (*i*.*e*. the larvae onto which the wasp oviposits): the host parasitised during training is a pest of stored products and thelytokous wasps should then be the most rewarded when laying an egg in that host species. We interpret the better decision making in arrhenotokous wasps as a sign of better learning skills in the context of host-seeking at a distance. This conclusion does not rule out potential effects of other processes, such as sensitivity to the stimulus, attention, motivation or propensity to explore [57]. Such a difference in information-processing ability has rarely been documented at the inter-population level, and to our knowledge, our work provides the first demonstration in parasitoids (see [14, 57] for birds, and [58] for bumblebees).

Contrary to the inter-population variability in memory retention observed in *Nasionia vitripennis* [59], memory lasted at least 51 h in both strains of *V*. *canescens*. Indeed, the extent to which the choice behaviour was modified did not vary with the time elapsed after training. This result is in contradiction with that of Thiel et al. [60] reporting that olfactory memory acquired in an oviposition context had vanished at 24 h in thelytokous wasps whilst it was detectable from 24 h in arrhenotokous wasps but not before. Procedural differences, such as the training odour used (FFH *vs*. geraniol) or the number of hosts available during training (100 *vs*. 2), could be cited to account for this discrepancies. Furthermore, some flaws in the protocol (*e*.*g*., low sample sizes, modalities missing in the control group) cast doubts on these findings. These points preclude comparing thoroughly these results and ours.

In our experiment, the absence of differences between strains up to 51 h does not mean that such differences do not exist. First, memory after 51 h could decay following different functions in both strains. Second, its molecular support could differ between strains; a 51 h memory can be protein-synthesis dependent or independent (*e*.*g*. [9, 10, 61, 62]). Third, the two strains may differ in the inter-trial time interval (ITI) necessary to form a given phase of memory (as in *e*.*g*. *Cotesia rubecula* and *C*. *glomerata*, [8]). At last, the number of training trials needed to form a given memory phase may also differ between strains.

Because both arrhenotokous and thelytokous *V*. *canescens* are time-limited, that is, they produce more eggs than they can lay within their lifetime [63, 64], they are expected to be rate-maximizers. Saving time during host-searching by learning environmental cues associated with host presence should then be selected for [36]. However, the higher travel requirements faced by arrhenotokous wasps in the field (see Introduction) makes host-searching at a distance much critical for them than for thelytokous wasps. Combined with the fact that outdoor host larvae should also be more difficult to find on the basis of the kairomone they produce, we expected arrhenotokous females to display a greater capacity to learn new environmental cues associated with hosts than thelytokous ones. Our results match this prediction and even if studying more populations is required to allow their generalisation to the whole *V*. *canescens* species, they support the litterature, which tends to find a relation between host distribution and learning and memory features in parasitoids (for *Cotesia glomerata* and *C*. *rubecula*: [20]; for *Nasonia vitripennis* and *Lariophagus distinguendus*: [65]; but see [60] for *V*. *canescens* and [66] for *Leptopilina heterotoma*). Our results in addition to previous ones [24, 27, 29, 32, 67–72] strongly suggest that the two strains are each adapted in behavioural, physiological and life history traits to different environments. These numerous studies strengthen the findings and interpretation of the present work. However we cannot exclude that the genetic divergence linked to the reproductive modes—which could themselves also result from the different selective pressures occurring in the different habitats–may also explain the difference in cognitive traits we observed.

The arrhenotokous wasps’ greater ability to use the learned information could also be linked to ecological differences between strains other than host distribution. First, according to the classification of Vet and Dicke [73], arrhenotokous wasps are generalist, and thelytokous wasps are specialists [69, 70]; learning of host associated cues is expected to be more frequent in generalist than in specialists [74]. Second, the variability and heterogeneity in the field may facilitate the evolution of better information-processing skills, including learning [75]. Finally, learning in the context of courtship is known to occur in other insects (*e*.*g*. male fruit flies: [76]), and male *V*. *canescens* were shown to use complex information to find mates [77, 78]. Under the hypothesis of the evolution of a general learning ability [69, 56], the enhancement of cognitive ability in the context of mating could facilitate the evolution of cognitive ability in other contexts, including host-searching. Obviously the higher genetic variability in sexuals [28] may also favour the evolution of these cognitives capacities.

We could not find differences in memory dynamics between strains, although it is thought that differences in ecological constraints shape memory dynamics [4, 10]. Specific ecological constraints of each preferential habitat do not seem either to select for different number of training trials to form memory [60]. The possibility of an absence of selective pressure in stable environments on learning abilities may also explain these results [79]: in such a case, thelytokous wasps would only exhibit ancestral abilities. This argument is supported by observation data in a 50-years lab reared *Lymnaea* strain, which was shown to have similar learning abilities as the wild strain [80].

Our results also raise the question of the adaptive value of a memory lasting more than 51 h. From an evolutionary perspective, as long as a stored information piece remains reliable, it should not be dropped from memory [13, 81]. In our protocol, wasps did not receive any information questioning the association between the training odourand host presence, which could explain the observed length of memory. Differences between both strains could lie in the susceptibility of individuals to contradictory information: since information reliability is expected to decrease faster in variable environments, arrhenotokous wasps should be selected for a higher sensibility to retroactive interference, or for enhanced reversal learning, a way to forget out-of-date information, than thelytokous ones [82].

Our comparative approach is a contribution designed to address both ultimate and proximate questions regarding the evolution of cognitive abilities. It echoes the call for an increase in the number of experimental studies and species considered in cognitive ecology to reinforce the bridge between ecology and cognition [83].

## Supporting information

S1 Dataset Data on training and testing of each individual wasp. (XLSX)

S1 Supporting information Effect of oviposition and kairomone on the wasps’ choice behaviour. (DOCX)
