# Supplementary material for: Cognitive adaptation in asexual and sexual wasps living in contrasted environments
Source: PLoS One. 2017 May 12;12(5):e0177581. doi: 10.1371/journal.pone.0177581 (PMC5428991; doi:10.1371/journal.pone.0177581)
Supplement: S1 Supporting information — (DOCX) [file pone.0177581.s002.docx]

Supporting information

Effect of oviposition and kairomone on the wasps’ choice behaviour

The aim of this experiment was to test whether the behavioural plasticity observed in trained individuals could be attributed to an effect of oviposition and host kairomone encounter. We compared the choice behaviour of wasps submitted to two different treatments.

Trained wasps were allowed to oviposit in the presence of FFH solution (*i.e.* training odour) and host kairomone (see Material & Methods, Wasps’ training procedure). Using the same experimental design, control wasps (hereafter called “oviposition” wasps) were allowed to oviposit in the presence of host kairomone but without the training odour.

The choice behaviour of trained and oviposition wasps towards the training odour *vs.* no odour was tested 2 h or 26 h after training (see Materials & Methods, Test session) in both wasp strains (arrhenotokous and thelytokous).

The wasps used originated from the same populations as described in the Material and Methods, Biological Material section. The experiment was performed between November 2010 and February 2011.

|  | arrhenotokous wasps | | thelytokous wasps | |
| --- | --- | --- | --- | --- |
|  | trained | oviposition | trained | oviposition |
| *N* | 42 | 41 | 40 | 40 |
| % | 67% | 36% | 65% | 25% |
| Chi square test | $\chi_{1}^{2}$ = 6.36, *P* = 0.01 | | $\chi_{1}^{2}$= 11.36, *P* < 0.001 | |
